# Antibody-dependent-cellular-cytotoxicity-inducing antibodies significantly affect the post-exposure treatment of Ebola virus infection

**DOI:** 10.1038/srep45552

**Published:** 2017-03-30

**Authors:** Qiang Liu, Changfa Fan, Qianqian Li, Shuya Zhou, Weijin Huang, Lan Wang, Chunyun Sun, Meng Wang, Xi Wu, Jian Ma, Baowen Li, Liangzhi Xie, Youchun Wang

**Affiliations:** 1 Division of HIV/AIDS and Sex-transmitted Virus Vaccines, National Institutes for Food and Drug Control, Beijing 100050, China; 2 Division of Animal Model Research, Institute for Laboratory Animal Resources, National Institutes for Food and Drug Control, Beijing 100050, China; 3 Division of Monoclonal Antibody, National Institutes for Food and Drug Control, Beijing 100050, China; 4 Sino Biological Inc., Beijing 100176, China

## Abstract

Passive immunotherapy with monoclonal antibodies (mAbs) is an efficacious treatment for *Ebola virus* (EBOV) infections in animal models and humans. Understanding what constitutes a protective response is critical for the development of novel therapeutic strategies. We generated an EBOV-glycoprotein-pseudotyped Human immunodeficiency virus to develop sensitive neutralizing and antibody-dependent cellular cytotoxicity (ADCC) assays as well as a bioluminescent-imaging-based mouse infection model that does not require biosafety level 4 containment. The *in vivo* treatment efficiencies of three novel anti-EBOV mAbs at 12 h post-infection correlated with their *in vitro* anti-EBOV ADCC activities, without neutralizing activity. When they were treated with these mAbs, natural killer cell (NK)-deficient mice had lower viral clearance than WT mice, indicating that the anti-EBOV mechanism of the ADCC activity of these mAbs is predominantly mediated by NK cells. One potent anti-EBOV mAb (M318) displayed unprecedented neutralizing and ADCC activities (neutralization IC_50_, 0.018 μg/ml; ADCC EC_50_, 0.095 μg/ml). These results have important implications for the efficacy of antiviral drugs and vaccines as well as for pathogenicity studies of EBOV.

*Ebola virus* (EBOV), an enveloped, non-segmented negative-strand RNA virus of the family *Filoviridae*, causes severe haemorrhagic fever in humans and non-human primates, with fatality rates of up to 90%^1^,^2^. There are five species that have been identified: *Zaire ebolavirus, Sudan ebolavirus, Taï Forest ebolavirus, Bundibugyo ebolavirus*, and *Reston ebolavirus* species^3^. The virus that caused the 2014–15 West African outbreak belongs to the *Zaire ebolavirus* species. This was the first EBOV outbreak to last over 1 year and to spread to more than several hundred individuals^4^. Although many attempts have been made to treat EBOV infections, presently there are no licensed therapeutic agents for humans^5^, so an approach that directly clears infected cells is highly desirable as an effective treatment for EBOV infection.

Qiu and Wong *et al*. have highlighted IgG antibody levels as the major immune correlate of protection against EBOV infection^6^,^7^. In recent years, monoclonal antibody (mAb) cocktails have demonstrated high efficacy in the treatment of EBOV infections in humans and animal models, including ZMAb, MB-003, ZMapp, and MIL77^8^,^9^,^10^,^11^,^12^. However, although some of these anti-EBOV antibodies are neutralizing, others are non-neutralizing *in vitro* but still provide *in vivo* protection against EBOV, suggesting that their anti-EBOV effect is due to a protective mechanism other than neutralization activity^13^,^14^. Notably, Corti *et al*. suggested that the specific neutralization mechanism of the mAb114 antibody and its *in vitro* antibody-dependent cellular cytotoxicity (ADCC) activity may contribute to its capacity to protect against lethal Ebola virus disease in macaques^15^. The goals of this study were to test the potential efficacy of four murine mAbs in the pre-exposure prevention and post-exposure treatment of EBOV pseudovirus infection and to address the fundamentally important question of whether or not ADCC-inducing antibodies are important in EBOV clearance after exposure.

The various infectious forms of EBOV, including the wild-type isolate^16^, recombinant EBOV^17^, and a mouse-adapted strain^18^, must be handled in a stringent biosafety level 4 (BSL-4) laboratory, which has created a bottleneck in the development of antiviral therapeutic agents. Recently, some pseudoviral-particle neutralization assays based on the luciferase reporter system have been reported that allow the evaluation of anti-EBOV agents in laboratories with a lower biosafety level of containment (BSL-2)^19^,^20^. The EBOV glycoprotein (GP) is solely responsible for viral attachment to, fusion with, and entry into new host cells, and it is therefore a major target of vaccine and drug design efforts^21^,^22^. Here, to facilitate the investigation of the critical events in viral infection and to identify the antiviral activity of neutralizing and ADCC-inducing antibodies, we generated a replication incompetent GP_1,2_-pseudotyped virus expressing the Fluc reporter protein (pHIV–ZGP–Fluc) using an envelope-defective strain of HIV-1 (strain SG3) as a way of safely and sensitively monitoring viral infections in cells and mice. These pseudovirus-infected mice are ideal models in which to evaluate antiviral efficiency *in vivo* in less time than is required for replication-competent EBOV assays.

With bioluminescence imaging (BLI) methods, we demonstrated the high treatment efficacy of novel murine mAbs (M401 and M318), which induced complete clearance of infected cells in all five mice in each group, even 12 h after challenge, and we located their binding epitopes in the receptor-binding domain of GP_1_. The efficacy of the post-infection treatments correlated with the *in vitro* anti-EBOV ADCC activities of the mAbs, without neutralization activity. Lastly, the transfer of either neutralizing or non-neutralizing mAbs to NK-deficient mice caused a lower level of viral clearance following challenge compared with that in similarly treated wild-type BALB/c mice, indicating that NK-mediated ADCC activity has an important role in this anti-EBOV treatment *in vivo*.

## Results

### Generation of the pseudotyped Ebola virus

**Figure 1:**
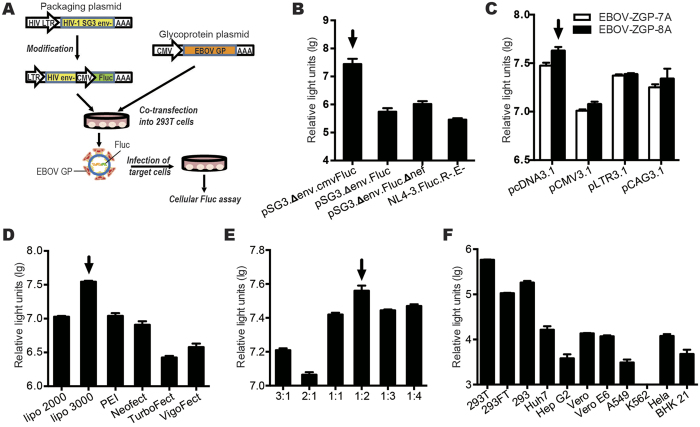
pHIV–ZGP–Fluc construction and cell sensitivity. (**A**) Procedural flow chart of pHIV–ZGP–Fluc construction. 293T cells were co-transfected with pEBOV-ZGP and pSG3.Δenv.cmv.Fluc. After 48 h, pHIV–ZGP–Fluc was collected from the supernatant, concentrated, purified, and used to infect the target cells. pHIV–ZGP–Fluc-infected cells were incubated for 48 h before the luciferase activity assay. (**B**) Optimization of the HIV framework plasmid. pEBOV-ZGP and different framework plasmids were co-transfected to generate pHIV–ZGP–Fluc. (**C**) Optimization of the GP expression vector. pSG3.Δenv.cmv.Fluc and different expression vectors (EBOV-ZGP-7A or EBOV-ZGP-8A) were co-transfected to generate pHIV–ZGP–Fluc. (**D**) Optimization of transfection reagents. pCDNA3.1–EBOV-ZGP-8A and pSG3.Δenv.cmv.Fluc were co-transfected with different transfection reagents. (**E**) Optimization of the proportion of plasmids. Different proportions of pCDNA3.1–EBOV-ZGP-8A and pSG3.Δenv.cmv.Fluc were tested. (**F**) Cell tropism of pHIV–ZGP–Fluc. Different cell lines were infected with pHIV–ZGP–Fluc. The relative light units of the infected cells were measured.

EBOV-GP_1,2_-pseudotyped HIV was generated by the co-transfection of human embryonic kidney (HEK) 293T cells with a lentiviral vector and a EBOV-GP_1,2_-expressing construct (Fig. 1A). To obtain a high titre of pHIV–ZGP–Fluc, we modified and optimized four parameters that could influence the generation of the pseudotyped virus, including the HIV framework plasmid, the glycoprotein expression vector, the transfection reagents, and the relative proportions of the EBOV GP expression plasmid and the HIV framework plasmid. When modifying and selecting the packaging plasmid and the GP expression plasmid, four HIV framework plasmids (Fig. 1B) and four expression plasmids with two *GP* genes, EBOV-ZGP-7A or RNA-edited EBOV-ZGP-8A (Fig. 1C), were used to construct the pseudoviruses, among which pSG3.Δenv.cmv.Fluc and pCDNA3.1– EBOV-ZGP-8A was found to be the best combination. A series of transfection reagents was tested to maximize the titre of the constructed pseudovirus, and Lipofectamine 3000 produced the highest transfection efficiency (Fig. 1D). To maximize the pseudovirus yield, the ratio of the two plasmids used for transfection, pCDNA3.1–EBOV-ZGP-8A and pSG3.Δenv.cmv.Fluc, was optimized to 1:2 (Fig. 1E). Finally, we tested the potential cellular tropism of pHIV–ZGP–Fluc with various cell lines. Most of the cell lines that we analysed were permissive to pHIV–ZGP–Fluc infection, demonstrating the wide cellular tropism of pseudotyped EBOV. However, HEK293T cells showed the highest infection efficiency (Fig. 1F).

### Constructing a mouse model of pHIV–ZGP–Fluc infection

Different strains of mice, including KM, NIH, BALB/c, and C57BL/6 mice, were infected with 1 × 10^7^ median tissue culture infective doses (TCID_50_) of pHIV–ZGP–Fluc by intraperitoneal (IP) injection to establish an appropriate mouse model of Ebola infection. The pseudotyped HIV virus was able to bind and enter into cells *in vivo* via the interaction between the EBOV GP and the receptor on the host cell surface, after which infected cells expressed the Fluc reporter protein. Following the addition of D-luciferin, a substrate of Fluc, the resulting bioluminescence light could be acquired with an Imaging System. At 4 days post-infection (dpi), we anaesthetized and observed the live mice with a BLI method.

**Figure 2:**
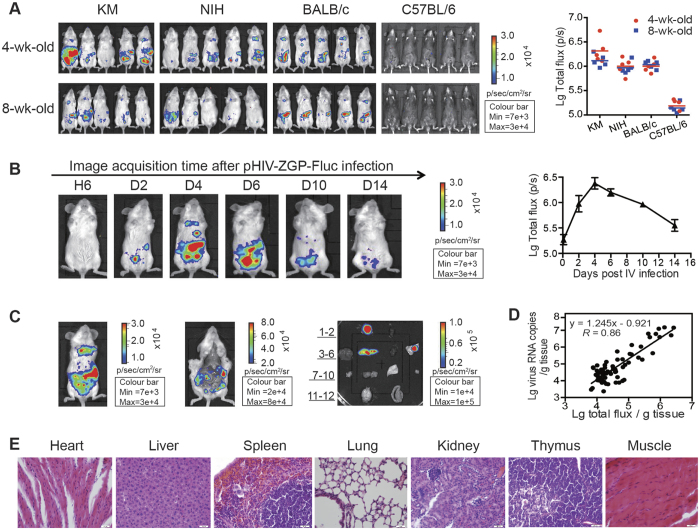
Construction and identification of a mouse model of pHIV–ZGP–Fluc infection. (**A**) Four-week-old and 8-week-old female KM, NIH, BALB/c, and C57BL/6 mice were inoculated with pHIV–ZGP–Fluc by IP injection (1 × 10^7^ TCID_50_/mouse). The relative levels of bioluminescence are shown in pseudocolours, with red and blue representing the strongest and weakest photon fluxes, respectively. Values of total flux for each group at 4 dpi are shown on the right. (**B**) BALB/c mice were inoculated with pHIV–ZGP–Fluc and monitored for the duration of bioluminescence. Bioluminescent images were superimposed on grey-scale photographs at 6 h, 2 days, 4 days, 6 days, 10 days, and 14 days post-infection. Values for total flux at different time points are shown on the right. Each data point is a mean value (n = 5). (**C**) Photon fluxes in infected BALB/c mice and their dissected tissues were measured at 4 dpi. Different organs and tissues shown are 1) thymus, 2) heart, 3) liver, 4) spleen, 5) kidney, 6) lung, 7) lymph node, 8) muscle, 9) skin, 10) ovary, 11) brain, and 12) intestine. (**D**) Correlation of pHIV–ZGP–Fluc viral loads in various tissues with bioluminescence intensity at 4 dpi. (**E**) Histopathological analysis of heart, liver, spleen, lung, kidney, thymus, and muscle at 7 dpi. Paraffin-fixed tissue sections were stained with haematoxylin and eosin. Arrows indicate lesion sites. Scale bar, 20 μm.

When bioluminescence was detected in the chests and abdomens of the KM, NIH, and BALB/c mice, the greatest levels of homogeneity were observed in the 4-week-old and 8-week-old BALB/c mice (Fig. 2A). Therefore, we used BALB/c mice in our BLI experiments to monitor the progression and duration of pHIV–ZGP–Fluc infection. The viral construct pHIV–ZGP–Fluc was then titrated *in vivo*. Four groups of mice (n = 20) were inoculated by IP injection with different doses of the pHIV–ZGP–Fluc virus (500 μl, 50 μl, or 5 μl; 1 × 10^7^ TCID_50_ per ml). The pHIV–ZGP–Fluc virus titration in BALB/c mice was determined to be 60 50% animal infectious dose (AID_50_) per ml (Supplementary Fig. 1). After IP infection with 60 AID_50_, the luciferase gene contained in pHIV–ZGP–Fluc was first expressed at 2 dpi, peaked at 4 dpi, and remained detectable for at least 14 days after the initial infection (Fig. 2B). Various tissues from the infected mice were imaged at 4 dpi. Photon flux was detected in the thymus, spleen, lung, and mesentery (Fig. 2C). To determine whether or not there was a direct correlation between the bioluminescence intensity and the viral titre in these tissues, we collected the tissues from mice at 4 dpi and measured both the luciferase units per gram of tissue, using BLI, and the virus RNA copies per gram of tissue, using quantitative reverse transcription (RT)–PCR. A good correlation (R = 0.86) was observed between the bioluminescence intensity and the viral load, confirming that bioluminescence is a useful marker for assessing the viral load in the tissues of mice infected with pHIV–ZGP–Fluc (Fig. 2D). To assess the pathogenicity of pHIV–ZGP–Fluc in BALB/c mice, a systematic histopathological analysis of the heart, liver, spleen, lung, kidney, thymus, and muscle was performed. No histopathological changes were detected in any tissue dissected from the BALB/c mice at 7 dpi, which confirmed that pHIV–ZGP–Fluc is a safe surrogate for Ebola virus in the research context.

### *In vitro* neutralizing and ADCC activities of the mAbs

**Figure 3:**
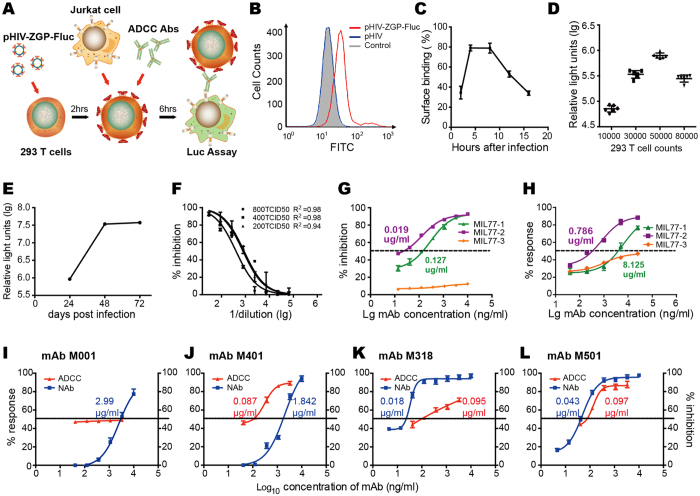
*In vitro* neutralizing and ADCC assays of human and murine anti-EBOV mAbs. (**A**) Procedural flow chart of the pHIV–ZGP–Fluc ADCC assay. (**B**) EBOV antigen presented on the cell surface was measured with flow cytometry. pHIV–ZGP–Fluc and pHIV (strain SF162) viruses were used to infect CEM cells. The non-virus group was used as a control, and antibody M401-FITC was used to detect the EBOV antigen. (**C**) The M401-FITC binding activity at different time points (2, 4, 8, 12, and 16 hpi) was measured as in (**B**). (**D**–**F**) Optimization of the pseudovirus-based neutralization assay. (**D**) Optimization of HEK293T cell density. The x-axis indicates different numbers of HEK293T cells. Dots show relative light units after incubation for 48 h. (**E**) Optimization of incubation time before detection. The x-axis indicates the incubation time, and the y-axis indicates the relative light units. (**F**) Dose–response between pHIV–ZGP–Fluc input and IC_50_ values of mAbs. Serial dilutions of mAbs directed against EBOV GP were pre-incubated with pHIV–ZGP–Fluc, and the percentage inhibition and R^2^ were determined. (**G**,**H**) *In vitro* neutralization activities (**G**) and ADCC activities (**H**) of the MIL77 monoclonal antibodies, MIL77–1, MIL77–2, and MIL77–3. Calculated IC_50_ and EC_50_ values are shown next to each curve. (**I**–**L**) *In vitro* neutralization and ADCC activities of four anti-EBOV-GP mAbs, M001 (**I**), M401 (**J**), M318 (**K**), and M501 (**L**). Calculated IC_50_ and EC_50_ values are shown next to each curve.

An *in vitro* ADCC assay was developed based on the GP_1,2_-pseudotyped virus. The conformational GP_1,2_ proteins presented on the surfaces of infected cells were targeted by the Jurkat-effector-cell-mediated ADCC response, which was induced by the tested mAbs (Fig. 3A). Using flow cytometry, we showed that the peak fluorescence level when the M401 antibody bound to pHIV–ZGP–Fluc-infected CEM cells was five-fold higher than that when it bound to pHIV (strain SF162)-infected cells, suggesting that many GP protein molecules were displayed on the pseudovirus-infected cell surfaces at 12 hours post-infection (hpi) (Fig. 3B). The binding activities peaked at 4 hpi and remained detectable until at least 16 dpi (Fig. 3C). Notably, in this newly developed ADCC assay, the pseudovirus containing a Fluc reporter gene was used to infect target cells, so the bioluminescence detected at 12 hpi resulted from expression by both this pseudovirus and the Jurkat effector cells, which stably express an NFAT-induced luciferase. We found that the percentage of bioluminescence produced by the pseudovirus was not higher than 0.3% of that produced by the Jurkat effector cells in response to ADCC, so there was no significant difference between the ADCC activities of mAbs measured with or without a pseudovirus expressing a luciferase reporter gene. We optimized the multiplicity of infection (MOI) and the ratio of effector and target cells to find the best conditions for this new assay. First, MOIs ranging from 0.01 to 2.00 were tested. There was little difference in the amount of mAb binding on target cells when the MOI was higher than 1.00, but there was an obvious improvement when the MOI was lower than 1.00. Similar optimization experiments were performed with various ratios of effector and target cells; these revealed that the best ratio was 10:1. With these optimized conditions, the ADCC of mAbs can be measured by our novel pseudovirus method.

We also established a high-throughput pseudovirus-based neutralizing antibody method, and the cell count, incubation time, and pseudovirus input were similarly optimized. The Fluc gene encoded by the pseudovirus backbone was expressed in infected cells and can be detected and measured. Notably, no Fluc signal should be detected if the pseudoviruses have been neutralized by neutralizing antibodies because this renders the pseudoviruses unable to infect cells. We determined that a cell density of 5,000 cells/well in a 96-well plate was the optimal density for this assay (Fig. 3D). We chose 48 h as the incubation time because the number of relative light units reached a plateau at that time (Fig. 3E). Finally, serum samples from a pCDNA3.1–EBOV-ZGP-8A-vaccinated guinea pig were used to identify the optimal viral input. The coefficients of determination were excellent with 200, 400, and 800 TCID_50_ of pHIV–ZGP–Fluc, so the medium viral input of 400 TCID_50_ was selected (Fig. 3F). Using this new neutralization assay, the potent human–mouse chimeric MIL77 antibodies MIL77-1, MIL77-2, and MIL77-3 were shown to have neutralization half-maximal inhibitory concentration (IC_50_) values of 0.127 μg/ml (95% CI, 0.080–0.174 μg/ml), 0.019 μg/ml (95% CI, 0.010–0.028 μg/ml), and undetectable, respectively (Fig. 3G). Additionally, the ADCC responses were detected for these three mAbs, resulting in respective half-maximal effective concentrations (EC_50_) of 8.125 μg/ml (95% CI, 4.575–11.675 μg/ml), 0.786 μg/ml (95% CI, 0.552–0.102 μg/ml), and undetectable (Fig. 3H). We also tested the neutralizing and ADCC-inducing activities of four murine mAbs, M001, M401, M318, and M501. M001 was negative for ADCC-inducing activity and had a low neutralization titre (Fig. 3I). M401 induced a potent ADCC response, with an EC_50_ of 0.087 μg/ml (95% CI, 0.052–0.122 μg/ml) (Fig. 3J). Notably, M318 and M501 each had high neutralizing activity (IC_50_ values of 0.018 μg/ml [95% CI, 0.013–0.023 μg/ml] and 0.043 μg/ml [95% CI, 0.029–0.057 μg/ml], respectively) and ADCC activity (EC_50_ values of 0.095 μg/ml [95% CI, 0.074–0.116 μg/ml] and 0.097 μg/ml [95% CI, 0.078–0.116 μg/ml], respectively) (Fig. 3K,L).

### *In vivo* prevention and treatment efficacies of human and murine mAbs

To assess the *in vivo* prevention efficacies of the MIL77 antibody cocktail (MIL77-1, MIL77-2, and MIL77-3), mice were administered two doses of the cocktail or of each mAb individually (5 mg/kg) before pseudovirus exposure (day −3 and −4 h) via the intravenous (IV) route and were then challenged with an IP injection of 60 AID_50_ of pHIV–ZGP–Fluc (day 0). Nearly complete protection was conferred by the MIL77 cocktail and by MIL77-1 or MIL77-2 alone (*p* < 0.001), whereas the bioluminescence was not significantly reduced from control levels in the MIL77-3-injected mice. However, no protection against pHIV–ZGP–Fluc infection was conferred when two doses of the cocktail (5 mg/kg) were administered after exposure (+12 h and +2 d, *p* = 0.07) (Supplementary Fig. 2).

**Figure 4:**
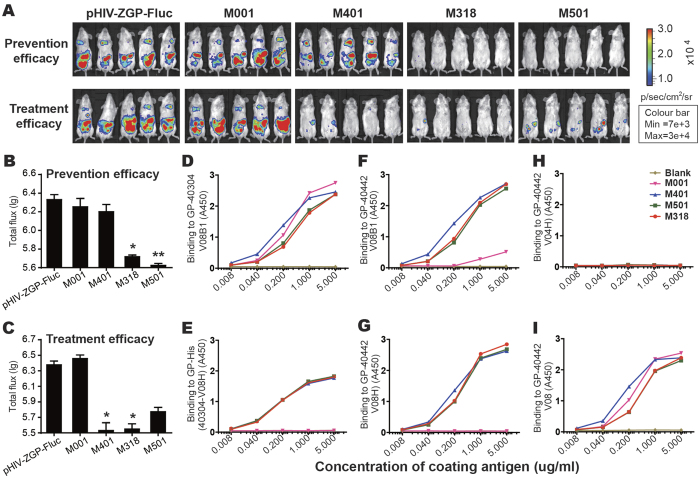
*In vivo* prevention and treatment efficacy assays. (**A**) Four murine anti-EBOV mAbs, M001, M401, M318, and M501, were injected to evaluate their prevention efficacies (upper) and treatment efficacies (lower). The mAbs were injected at 3 days or 4 h before the inoculation of pHIV–ZGP–Fluc to determine their preventive efficacies, and at 12 h or 3 days after viral inoculation to determine their treatment efficacies. Photon flux was measured at 4 dpi. The first column shows the control pHIV–ZGP–Fluc-infected BALB/c mice that did not receive any mAb treatment. (**B**–**C**) Preventive efficacy (**B**) and treatment efficacy (**C**) were detected as the total photon flux in pHIV–ZGP–Fluc-infected mice treated with various mAbs compared with that in similarly infected mice without mAb treatment (first column). *p < 0.05; **p < 0.01. (**D**–**I**) ELISA binding curves for recombinant GP proteins, including recombinant GP (**D**) and its receptor-binding domain (**E**) from EBOV strain H.sapiens-wt/GIN/2014/Kissidougou-C15 and recombinant GP (**F**), receptor-binding domain (**G**), GP2 (**H**), and GP1 (**I**) from EBOV strain Mayinga 1976.

The prevention and treatment efficacies of the four murine mAbs, M001, M401, M318, and M501, were evaluated with the same method (Fig. 4A). The photon flux was detected 4 dpi. The mice treated with either M318 or M518 before exposure to the pseudovirus had significantly less bioluminescence and infection levels that were 10-fold lower (*p* < 0.01) than untreated mice. However, hardly any protection was afforded by M001 or M401 (Fig. 4A upper, B). Next, to determine their treatment efficacies, these four mAbs (5 mg/kg per dose) were injected at 12 h and 2 days after pseudovirus inoculation. M318 not only had a high preventive efficacy, it also provided complete protection when applied post-exposure (*p* < 0.01); interestingly, the treatment activity of M401 was much higher than its preventive efficacy (Fig. 4A lower, C). The treatment efficiencies of these four mAbs at 12 hpi correlated well with their *in vitro* anti-EBOV ADCC-inducing activities, without neutralizing activity. Notably, unlike M401 and M318, the marked drop in bioluminescence following post-exposure treatment with M501 was not statistically significant (*p* = 0.07), even though the ADCC activities of these three mAbs were comparable. This result may have been caused by these three mAbs interacting with different types of effector cells *in vivo*. The treatment of mice with any of these three mAbs (M401, M318, and M501) protected the animals in a dose-dependent manner (*p* < 0.01), and these ADCC-inducing antibodies cleared pHIV–ZGP–Fluc by 50% at concentrations of 1.0 mg/kg, 2.3 mg/kg, and 2.0 mg/kg, respectively (corresponding to the whole immunoglobulin G molecule) (Supplementary Fig. 3). All four tested murine mAbs bound the GP and GP_1_ proteins, but not the GP_2_ protein (Fig. 4D,F,H,I). Three of these mAbs (M401, M318, and M501), but not M001, bound the receptor-binding domain (Fig. 4E,G), suggesting that the epitopes targeted by the antibodies that mediate ADCC overlap in the receptor-binding domain with those recognized by antibodies capable of viral neutralization.

### NK cells mediate ADCC against pseudovirus infection

**Figure 5:**
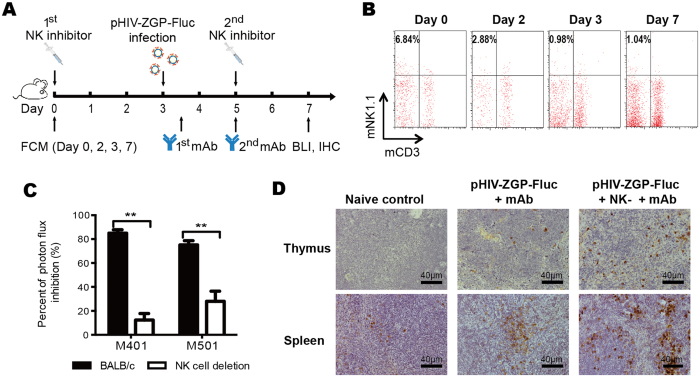
Identification of NK-cell-mediated ADCC against pHIV–ZGP–Fluc infection. (**A**) NK inhibitor, mAbs, and pHIV–ZGP–Fluc virus were injected as shown in the procedural flow chart. BALB/c mice were treated with the NK inhibitor at baseline and 5 days. The mice were infected with pHIV–ZGP–Fluc at 3 days and treated with mAbs at 12 h and 2 dpi. Bioluminescent images of BALB/c mice were analysed at 4 dpi with the IVIS system. (**B**) Percentage of NK cells in mouse blood. Flow cytometry was performed at 0, 2, 3, and 7 days to confirm the deletion of NK cells. mCD3^−^mNK1.1^+^ cells were recognized as NK cells, and the percentage of NK cells was calculated as shown. (**C**) Viral clearance rates mediated by mAbs M401 and M501 were evaluated after NK deletion compared with those in normal BALB/c mice. (**D**) Immunohistochemical staining of pHIV–ZGP–Fluc in thymus and spleen. Sections of BALB/c mice (naïve), pHIV–ZGP–Fluc-infected BALB/c mice treated with M401 (pHIV–ZGP–Fluc + mAb), and NK-deleted mice with the same treatment (pHIV–ZGP–Fluc + NK^−^ + mAb) were stained with monoclonal mouse anti-EBOV antibody. Scale bar, 20 μm.

The working principle for this kind of ADCC-inducing mAb *in vivo* is based on the interaction between the antibody and the GP located on the surfaces of infected cells. The antibodies mediate effector cell cytotoxicity by binding the Fcγ receptor, resulting in the clearance of the virus. To investigate the role of NK cells in ADCC mediation, experiments were performed as shown the schematic in Fig. 5A. BALB/c mice received two IV injections of NK inhibitor (50 μg per mouse, anti-asialo ganglio-N-tetraosylceramide [GM1]), separated by a 5-day interval, to repress the functions of NK cells. This significantly reduced the number of NK cells (CD3^−^/mNK1.1^+^) in the peripheral blood of the BALB/c mice from day 2 after the NK inhibitor was injected (2.88%) compared with that in the mice lacking an NK inhibition treatment (on day 0, 6.84%). The suppression of NK cells increased (day 3, 0.98%) and lasted until the end of the observation period (day 7, 1.04%) (Fig. 5B). Three days after the injection of the NK inhibitor, 60 AID_50_ of pHIV–ZGP–Fluc was inoculated into each mouse. The mAbs were then administered to the NK-deleted mice 12 h and 2 days after viral inoculation to evaluate the ADCC activity in the absence of NK cell function. At 4 dpi, the photon flux in the NK-suppressed mice was compared with that in untreated BALB/c mice. The inhibition of photon flux in NK-suppressed mice compared with that in mice that were only virus-infected represents the ADCC efficacy. Significantly lower mAb ADCC efficacies were observed when NK cells were deleted (*p* < 0.01), regardless of whether non-neutralizing M401 or neutralizing M501 was used, suggesting that the antiviral treatment mechanisms of NK cells are needed to mediate the ADCC induced by these antibodies. However, the reduction in ADCC efficiency was greater with M401 than with M501, indicating that the NK-cell-mediated ADCC induced by the M401 antibody is greater than that induced by the M501 antibody (Fig. 5C). The M401-treated mice were immunohistochemically stained for EBOV to confirm the mediation of ADCC by NK cells. The thymus and spleen tissues of the mice were analysed with immunohistochemistry based on the BLI observations. Consistent with the BLI results, more extensive EBOV staining was observed in the mice lacking NK cells, which suggests that the ADCC activity of some mAbs, such as M401 and M501, is closely associated with NK cell function.

## Discussion

The objective of the this study was use safe and sensitive cellular assays and a BLI mouse model infected with a EBOV-GP_1,2_-pseudotyed HIV virus to clarify the role of ADCC activity in controlling EBOV. Our results demonstrate that this pseudovirus-based system is suitable for evaluating the ADCC response *in vitro* and *in vivo* and that NK-cell-mediated ADCC plays a critical role in EBOV clearance. The working principle of this kind of ADCC-inducing mAb is based on the interaction between the antibody and the EBOV GP located on the surfaces of infected cells. The antibodies mediate NK cell cytotoxicity by binding to the Fcγ receptor, resulting in clearance of the virus. Because the pHIV–ZGP–Fluc pseudovirus has no *GP* gene, no new GP protein is expressed on the surfaces of the targeted HEK293T cells after infection. However, the conformational epitopes of the GP protein were strongly detected on the cell surfaces after infection, even at 12 h post-infection. We suggest that the EBOV surface GP protein is displayed on the infected cell surface by unknown pathway after GP protein binds to its receptor and the virus enters the cell via endocytosis.

The highly predictive value of this model is attributable, in part, to the high sensitivity of the BLI technology, which has been used to study various viral pathogens by detecting luminescence-tagged targets (luciferase or enhanced green fluorescent protein) in live animals^23^. BLI can identify unexpected infection sites and patterns that could be easily missed without real-time imaging^24^. In this mouse model of pseudovirus infection via IP injection, bioluminescence was detected 2 dpi, and high levels of the Fluc reporter protein were expressed in the mesentery, thymus, spleen, and lung at 4 dpi. As previously described in a live EBOV model, cells of the mononuclear phagocytic system are the earliest targets of infection. Viral replication was observed by 2 dpi in macrophages in the lymph nodes and spleen^25^. Good correlations were observed between the bioluminescence intensity, viral load, and immunohistochemical staining in various tissues in this study, suggesting that BLI can be used as an alternative way to evaluate the efficacy of anti-EBOV treatments.

EBOV GP is the only virally expressed protein on the virion surface and is critical for the attachment of the virus to host cells and the catalysis of membrane fusion. Until now, many candidate vaccines, neutralizing antibodies, and other antiviral agents specific to the GP protein have been assessed with mouse infection models using a lethal EBOV mouse-adapted virus^26^,^27^. Interestingly, inbred laboratory mice were resistant to infection with non-adapted EBOV^28^, but are permissive to infection with the GP-pseudotyped virus, which suggests that EBOV infection in rodents is not blocked at the step at which the virus binds to its receptor or at the membrane fusion step. GP-pseudotyped HIV has also clarified the role of GP in EBOV pathogenesis. The primary pathology of EBOV haemorrhagic fever is vascular injury and coagulation abnormalities, and GP has been implicated as the EBOV protein primarily responsible for cytotoxicity and vascular permeability^29^. However, no relative pathological changes were detected in mice 4 days after pseudoviral infection, suggesting that it is not the full-length GP_1,2_ protein that confers the lethal phenotype on this model^30^. The truncated surface GP, designated ‘shed GP’, released from virus-infected cells can activate uninfected dendritic cells and macrophages, causing a massive release of pro- and anti-inflammatory cytokines and vascular permeability^31^.

Notably, one mAb (M318) displayed unprecedented potency, with a neutralization IC_50_ value of 0.018 μg/ml and an ADCC EC_50_ value of 0.095 μg/ml, which is similar to the efficacy of the positive control MIL77-2 antibody, which has a neutralization IC_50_ value of 0.019 μg/ml. Its efficacy is also similar to the potent neutralizing antibody mAb114, which has an IC_50_ value of 0.09 μg/ml in the same pseudovirus neutralization cellular assay, a maximal ADCC activity of 0.03 μg/ml, and is considered to be the most potent neutralizing activity described so far^15^,^32^. This is the first study to define antibodies as either neutralizing or ADCC-inducing antibodies and to describe their use in anti-EBOV prevention or treatment strategies based on their *in vitro* neutralizing or ADCC-inducing properties. When we examined the relationship between or the overlap in the specificities of the antibodies that mediate these two antiviral activities, we observed that the ADCC activity specific to EBOV correlated with the binding of the antibodies to the GP on the surfaces of virus-infected cells, without viral neutralization, which constitute instances of ADCC in the absence of detectable neutralization, and *vice versa*. Surprisingly, nearly complete viral clearance was observed in the M318-injected mice, regardless of whether the mAb was injected pre-exposure or post-exposure to pseudoviral infection. Because the human–mouse chimeric MIL77 cocktail, which has potent neutralizing activity, was not effective in the treatment of pseudovirus-infected mice when injected at 12 hpi, we consider that the high treatment efficiencies observed in the M318- and M401-inoculated groups were attributable to the ADCC activities of these antibodies, regardless of whether or not they have high neutralization activity. The treatment efficacy of the MIL-77 cocktail could not be evaluated in our pseudovirus infection BALB/c model because there would be no cross-reaction between the human–mouse chimeric MIL-77 mAbs and the Fcγ receptor of the mouse NK cells, so further experiments with PBMC humanized mice should be conducted for an *in vivo* evaluation of the post-exposure efficacy of MIL77 components.

Three of the tested murine mAbs bound the receptor-binding domain of GP_1_, but not GP_2_. Notably, Zachary A. Bornholdt isolated 349 GP-specific mAbs from the peripheral B cells of a convalescent donor and characterized 77% of the mAbs as capable of neutralizing live EBOV. Several of these mAbs displayed unprecedented potency. Examination of the structures of selected mAbs in complexes with GP revealed a site of vulnerability located at the GP_1_–GP_2_ interface and the GP stalk region. Neutralizing antibodies targeting this site showed potent therapeutic efficacy against lethal EBOV challenge in mice^33^. However, the ZMapp mAbs, including C13C6, 2G4, and 4G7, are believed to exert the best preventive effects, and all three of these mAbs recognize conformational epitopes located on GP_2_ or the stem region of the GP trimer, whereas the remaining three antibodies from MB-003 and ZMAb bound to the trimer head^3^. Based on its recognition of distinct regions of GP, M318 could be used as part of a combination immunotherapy with ZMapp or a ZMapp-like MIL77 cocktail to maximize its efficacy.

The severity of EBOV infection and the lack of effective therapeutic solutions argue for sustained investment in all promising approaches to the prevention or treatment of this disease. Because ADCC plays an important role in protecting against initial infection, our data suggest that the development of ADCC-inducing antibodies or the addition of ADCC-enhancing mutations to neutralizing antibodies could augment their prophylactic and/or therapeutic immunotherapy potential against EBOV infection. In this study, we have found a way to correlate *in vivo* and *in vitro* ADCC activities evaluation, and the long-term goal is to aim at reducing the use of animals in research. However, the limitations of the pseudovirus infection model should also be taken into account, given that the pseudotyped HIV virus was replication incompetent, neutralization would have little effect in post-exposure treatment, suggesting that this pseudovirus infection model does not have the same constraints as the live target virus.

## Materials and Methods

### Cells

HEK293T (American Type Culture Collection [ATCC], CRL-3216), HEK293FT (Invitrogen, Carlsbad, CA, USA), HEK293 (ATCC, CRL-1573), HepG2 (ATCC, HB-8065),Vero (ATCC,CCL81),Vero E6 (ATCC, CRL-1586), A549 (ATCC, CCL-185), HeLa (ATCC, CCL-2), and BHK21 cells (ATCC, CCL-10) were grown in Dulbecco’s modified Eagle’s medium (HyClone, South Logan, UT, USA) supplemented with 10% foetal bovine serum (Gibco, Carlsbad, CA, USA), 1% penicillin–streptomycin solution (Gibco) and 2% 4-(2-hydroxyethyl)-1-piperazineethanesulfonic acid (Gibco) at 37 °C under 5% CO_2_. K-562 cells (ATCC, CCL-243) were grown in RPMI 1640 (HyClone) supplemented with 10% foetal bovine serum and 1% penicillin–streptomycin solution.

### Pseudotyped virus

A replication incompetent pseudotyped HIV (strain SF162) and HIV pseudotyped with GP_1,2_ of EBOV expressing the firefly luciferase reporter protein (Fluc) were generated as previously described^34^. In brief, the lentivirus-based pHIV–ZGP–Fluc construct carrying the EBOV glycoprotein *GP* gene was generated by the co-transfection of 293T cells with pCDNA3.1–EBOV-ZGP-8A and pSG3.Δenv.cmv.Fluc in a 1:2 ratio, using Lipofectamine 3000 (Invitrogen). After incubation for 48 h, the culture supernatant was centrifuged at 210 × *g* for 5 min, filtered through a 0.45 μM pore-size filter, and concentrated with a 30-kDa ultrafiltration centrifugal tube (Millipore, Boston, MA, USA). All works involving pseudotyped EBOV were performed in a BSL-2 facility at the National Institutes of Food and Drug Control, Beijing, China.

### Murine and human mAbs

A 6-week-old female BALB/c mouse was immunized by an IP injection with 5 μg of a recombinant GP1 protein (40442-V08B) from EBOV (strainH.sapiens-wt/GIN/2014/Kissidougou- C15) and recombinant GP protein (40304-V08B1) from subtype Zaire, strain Mayinga 1976 (Sinobiological Inc., Beijing, China). RNA extracted from its spleen cells was used to establish an immunized phage display antibody library^35^. In brief, antibodies that specifically bound the GP protein were isolated after four rounds of panning, and the variable region of the light chain or heavy chain of these antibodies was then ligated to the mouse constant region of the kappa light chain or IgG1 separately, using PCR technology. The mouse antibodies were transiently expressed in HEK293 cells. Three murine mAbs, M318, M401, and M501, were obtained from the GP-immunized library of EBOV H.sapiens-wt/GIN/2014/Kissidougou-C15, and M001 was obtained from the GP-immunized library of Zaire strain Mayinga 1976. For the enzyme-linked immunosorbent assay (ELISA) of antibody binding, a recombinant GP protein (40442-V08B1) from EBOV (strain H.sapiens-wt/GIN/2014/Kissidougou-C15) and its recombinant GP receptor-binding domain (40442-V08H), recombinant GP1 protein (40442-V08B), recombinant GP2 protein (40442-V04H), and recombinant GP protein (40304-V08B1) from subtype Zaire, strain Mayinga 1976, and its recombinant GP receptor-binding domain (40304-V08H) were coated onto ELISA plates in serial five-fold dilutions (original concentration, 5 μg/ml). Mouse anti-GP antibody (2 μg/ml) and horseradish-peroxidase-labelled goat anti-mouse IgG Fc polyclonal antibody (0.5 μg/ml) were then added, and the optical density was read at 450 nm (OD_450_). MIL77, a ZMapp-like mAb cocktail, was produced in modified Chinese hamster ovary (CHO) cells, as previously described^10^, and it includes MIL77-1 (containing the variable regions of c2G4), MIL77-2 (containing the variable regions of c4G7), and MIL77-3 (containing the variable regions of C13C6). The framework regions of the antibodies in MIL77 were also modified to be more similar to human framework regions. The CHO cells used to express MIL77 were engineered to prevent fucosylation, which is similar to the N-glycosylation present in plant-produced ZMapp.

### *In vitro* neutralization and ADCC tests with pHIV–ZGP–Fluc

As in the high-throughput *in vitro* neutralization assay, pHIV–ZGP–Fluc was incubated with each mAb for 1 h at 37 °C and then mixed with HEK293T cells in a 96-well plate and incubated for 48 h. The infectivity of pHIV–ZGP–Fluc was determined by measuring the bioluminescence, as described previously^36^.

The ADCC activities of the mAbs were determined with a new method based on the pseudotyped virus, pHIV–ZGP–Fluc. First, 5,000 CEM cells/well were combined with the pseudotyped virus (MOI = 1), mixed well, and centrifuged at 1,200 × *g* for 2 h at room temperature. After incubation for 4–6 h, the infected CEM cells were washed thoroughly to remove both dead cells and uninfected virus, and then used as the target cells. MAbs against GP were serially diluted from 100 μg/mL to 10 ng/mL and were then added into each well along with 50,000 Jurkat cells, followed by a 4–6 h killing incubation. Jurkat cells can be stimulated by mAb-recognized target cells, 4–6 h after which they express Fluc. The ADCC activities of the mAbs were measured with the Promega ADCC Reporter Bioassay (Promega, G7102, Madison, WI, USA).

### Animal experiments

The mice used in this study were housed and handled strictly in accordance with the guidelines set by the Association for the Assessment and Accreditation of Laboratory Animal Care (Frederick, MD, USA). The study protocol was approved by the Animal Care and Use Committee at the National Institute for Food and Drug Control (NIFDC, Beijing, China). Four-week-old and 8-week-old BALB/c, C57BL/6, KM (an outbred mouse strain derived from Swiss mice), and NIH mice (an outbred mouse strain bred by National Institutes of Health) were obtained from the Institute for Laboratory Animal Resources, NIFDC. Four-week-old BALB/c mice were infected with a 60 AID_50_ dose of pHIV–ZGP–Fluc via the IP route and monitored for bioluminescent signals at different time points.

### Viral load assay

Viral loads were quantified with real-time RT–PCR. RNA was isolated and reverse transcribed with the reverse transcription (RT) PCR kit (Takara, Shiga, Japan) according to the manufacturer’s protocol. Quantitative real-time RT–PCR was performed with a TaqMan probe (Sinogenomax, Beijing, China) by using a LightCycler 480 Real-Time PCR System (Roche, Basel, Switzerland). The probe sequence was 5′-FAM-ACAGGAAACAGCAGCCAGGTCAGCCGA-TAMRA-3′. The primer sequences were forward: 5′-AGCACAGCAAGCAGCAGC-3′, and reverse: 5′-AGCACAGCAAGCAGCAGC-3′.

### BLI analysis

Bioluminescence was analysed with the IVIS Lumina Series III Imaging System (Xenogen, Baltimore, MD, USA) with image acquisition and analysis methods that have been described previously^37^. In brief, mice were anaesthetized with pelltobarbitalum natricum (240 mg/kg body weight) via the IP route, and luminescence was measured 10 min after an IP injection of the substrate, d-luciferin (50 mg/kg body weight; Xenogen-Caliper Corp., Alameda, CA, USA). The Living Image software (Caliper Life Sciences, Baltimore, MD, USA) was used to measure the luciferase activities, and the signals emitted from different regions of interest in the body were measured and presented as total fluxes, in photons/s. All data are presented as mean values ± SEM.

### Flow cytometry

Flow cytometry was used to measure the amount of viral envelope protein present on the infected cellular surfaces. Anti-EBOV and anti-HIV antibodies were all diluted to 10 μg/ml and incubated separately with the target cells at room temperature for 30 min. After the cell samples were washed twice in PBS for 5 min each with centrifugation at 300 × *g*, the secondary antibody (fluorescein isothiocyanate [FITC]-conjugated goat anti-mouse IgG antibody; CWBiotech, Beijing, China) was added, and the cells were washed again with PBS. The cells were then loaded onto a FACSCalibur flow cytometer (BD, Franklin Lakes, NJ, USA) according to the manufacturer’s instructions. The results were analysed with FlowJo 7.6.1 by gating on the FITC-positive cells. The inhibition of NK cells was measured as the ratio of mNK1.1-positive to mCD3-negative cells in the whole-blood lymphocytes of BALB/c mice. Anti-mNK1.1–phycoerythrin (BD, 557391) and anti-mCD3–FITC antibodies (BD, 553062) were added, and the red blood cells were then lysed with ACK lysis buffer (Invitrogen), washed with PBS, and incubated with BALB/c mouse whole blood harvested from the inner canthal orbital vein. The results were analysed by gating on the FITC-negative cells.

### Immunohistochemistry

Tissues, including the heart, lung, liver, spleen, kidney, intestine, thymus, skin, muscle, and brain, were dissected from the mice on day 4 after infection, fixed in 10% neutral-buffered formalin, embedded in paraffin, and sectioned (2-μm thickness). An immunohistochemical analysis was performed with a monoclonal mouse anti-EBOVA antibody (clone M401, diluted 1:300; kindly provided by Sino Biological Inc., Beijing, China), as previously described^38^.

### Statistical analysis

All graphs were generated with Prism 5.0 software (GraphPad, San Diego, CA, USA). Statistical significance was compared with a non-parametric one-way ANOVA or Student’s *t*-test. All *p*-values of <0.05 were considered statistically significant.

## Additional Information

**How to cite this article:** Liu, Q. *et al*. Antibody-dependent-cellular-cytotoxicity-inducing antibodies significantly affect the post-exposure treatment of Ebola virus infection. *Sci. Rep.* **7**, 45552; doi: 10.1038/srep45552 (2017).

**Publisher's note:** Springer Nature remains neutral with regard to jurisdictional claims in published maps and institutional affiliations.

## Supplementary Material

Supplementary Figures
