# Supplementary material for: Antibody-dependent-cellular-cytotoxicity-inducing antibodies significantly affect the post-exposure treatment of Ebola virus infection
Source: Sci Rep. 2017 Mar 30;7:45552. doi: 10.1038/srep45552 (PMC5372081; doi:10.1038/srep45552)
Supplement: Supplementary Figures [file srep45552-s1.pdf]

Antibody-dependent-cellular-cytotoxicity-inducing antibodies significantly affect the post-exposure treatment of Ebola virus infection

Qiang Liu[1]*, Changfa Fan[2]*, Qianqian Li[1]*, Shuya Zhou[2], Weijin Huang[1], Lan Wang[3], Chunyun Sun[4], Meng Wang[1], Xi Wu[2], Jian Ma[1], Baowen Li[2], Liangzhi Xie[4], Youchun Wang[1]

[1]Division of HIV/AIDS and Sex-transmitted Virus Vaccines, National Institutes for Food and Drug Control, Beijing 100050, China

[2]Division of Animal Model Research, Institute for Laboratory Animal Resources, National Institutes for Food and Drug Control, Beijing 100050, China

[3]Division of Monoclonal Antibody, National Institutes for Food and Drug Control, Beijing 100050, China

[4]Sino Biological Inc., Beijing 100176, China

*These authors contributed equally to this work.

Correspondence: Youchun Wang, MD, PhD, National Institutes for Food and Drug Control, No. 2 Tiantanxili, Beijing 100050, China. Tel.: +86 10 67095921; Fax: +86 10 67095795; E-mail: wangyc@nifdc.org.cn

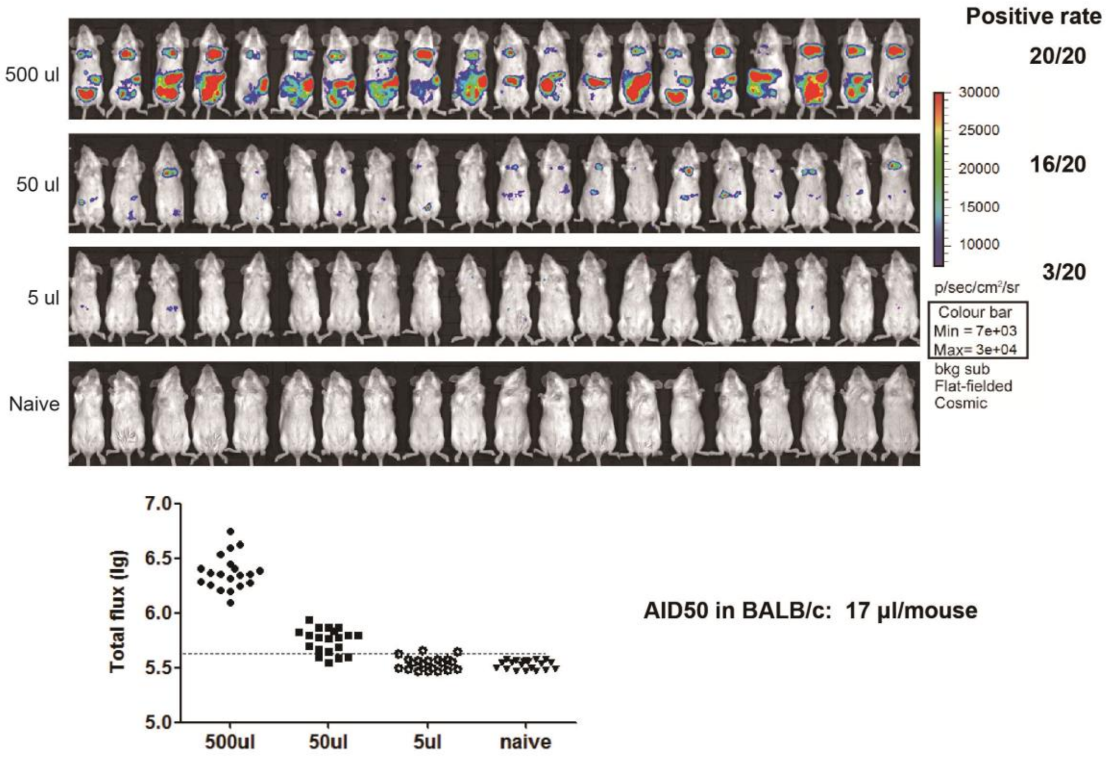

Supplementary Figure 1. Determination of 50% animal infectious dose (AID_{50}) of pHIV–ZGP–Fluc in BALB/c mice. BALB/c mice were inoculated with 500, 50, or 5 µl of pHIV–ZGP–Fluc virus (1×10^7 $TCID_{50}$ per ml) per mouse *via* the intraperitoneal route (n = 20). Total flux was analysed at 4 dpi. Positive infection rates are shown on the right. AID_{50} of pHIV–ZGP–Fluc in BALB/c mice was determined with the Reed–Muench method.

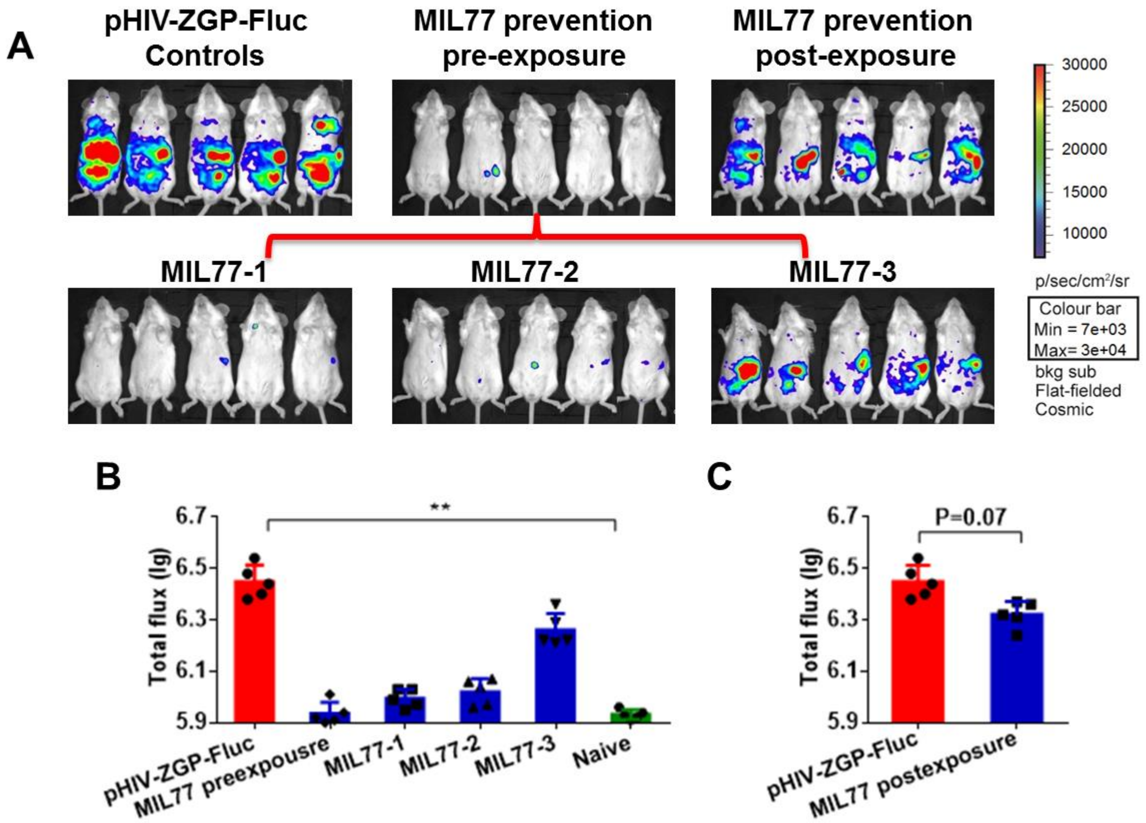

Supplementary Figure 2. Preventive efficacies of MILL77 antibodies in mice before and after exposure to the pseudovirus *in vivo*. A, MILL77 monoclonal antibodies and cocktail were administered before (−3 days and −4 h) and after (12 h and 2 days) pHIV–ZGP–Fluc infection. pHIV–ZGP–Fluc-infected mice without antibody inoculation were used as controls. Bioluminescent images were acquired at 4 dpi. B–C, Preventive efficacies were calculated as total flux values. Statistically significant differences (P values) are shown above. Significant preventive efficacies were detected in the MILL77 monoclonal antibody and cocktail pre-exposure groups (B), but not in the MILL77 cocktail post-exposure group (C). ** indicates extremely significant difference.

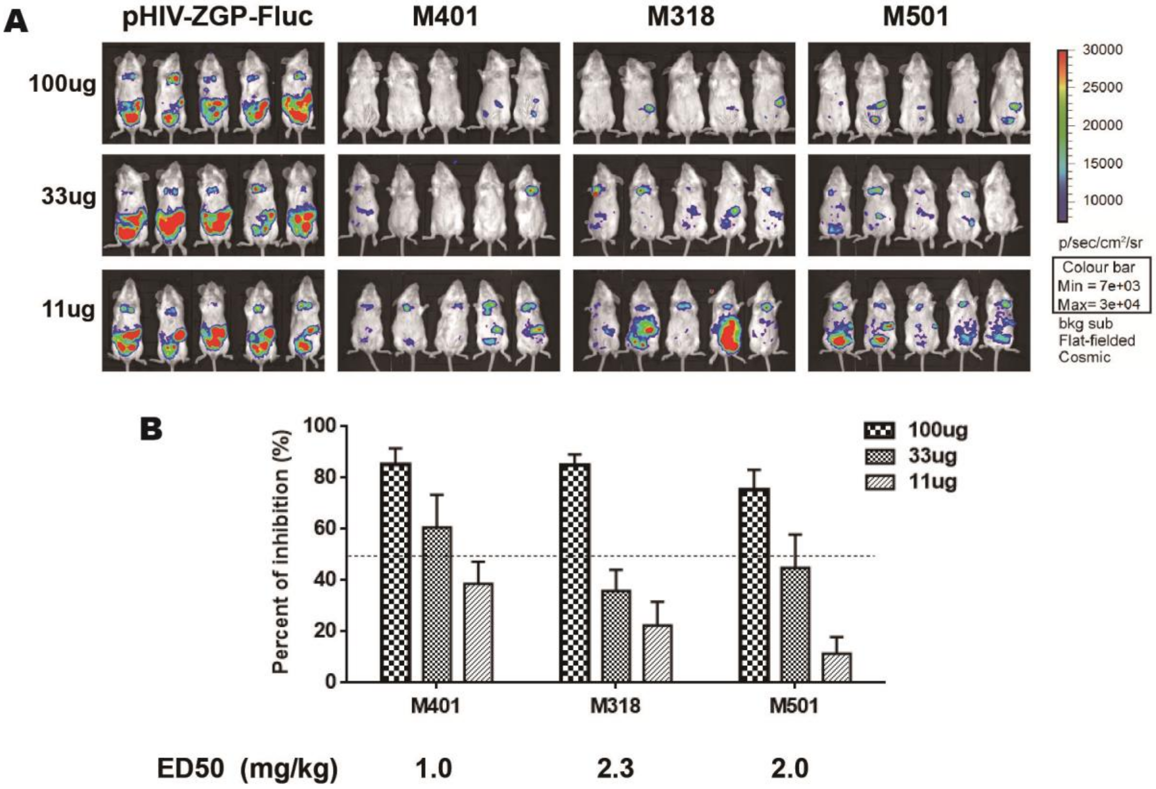

Supplementary Figure 3. Antibodies dose-dependently induce ADCC. (A) Mice were treated twice (at 12 h and 2 days post-infection) with each mAb (M401, M318, or M501) in graduated doses (100, 33, or 11 μg per dose). pHIV–ZGP–Fluc-infected mice without mAb treatment were used as controls. Bioluminescent images were acquired at 4 dpi. (B) The 50% effective doses (ED_{50}) of the mAbs that induced ADCC were calculated to be 1.0, 2.3, and 2.0 mg/kg, respectively, according to the inhibition of the total flux (shown at the bottom).
